# Identification of sero-reactive antigens for the early diagnosis of Johne’s disease in cattle

**DOI:** 10.1371/journal.pone.0184373

**Published:** 2017-09-01

**Authors:** Lingling Li, John P. Bannantine, Joseph J. Campo, Arlo Randall, Yrjo T. Grohn, Robab Katani, Megan Schilling, Jessica Radzio-Basu, Vivek Kapur

**Affiliations:** 1 Department of Animal Science, The Pennsylvania State University, University Park, PA, United States of America; 2 Huck Institutes of Life Sciences, The Pennsylvania State University, University Park, PA, United States of America; 3 National Animal Disease Center USDA-ARS, Ames, IA, United States of America; 4 Antigen Discovery, Inc., Irvine, CA, United States of America; 5 Cornell University, Ithaca, NY, United States of America; Universita degli Studi di Sassari, ITALY

## Abstract

*Mycobacterium avium* subsp. *paratuberculosis* (MAP) is the causative agent of Johne’s disease (JD), a chronic intestinal inflammatory disease of cattle and other ruminants. JD has a high herd prevalence and causes serious animal health problems and significant economic loss in domesticated ruminants throughout the world. Since serological detection of MAP infected animals during the early stages of infection remains challenging due to the low sensitivity of extant assays, we screened 180 well-characterized serum samples using a whole proteome microarray from *Mycobacterium tuberculosis* (MTB), a close relative of MAP. Based on extensive testing of serum and milk samples, fecal culture and qPCR for direct detection of MAP, the samples were previously assigned to one of 4 groups: negative low exposure (*n* = 30, NL); negative high exposure (*n* = 30, NH); fecal positive, ELISA negative (*n* = 60, F+E-); and fecal positive, ELISA positive (*n* = 60, F+E+). Of the 740 reactive proteins, several antigens were serologically recognized early but not late in infection, suggesting a complex and dynamic evolution of the MAP humoral immune response during disease progression. Ordinal logistic regression models identified a subset of 47 candidate proteins with significantly different normalized intensity values (p<0.05), including 12 in the NH and 23 in F+E- groups, suggesting potential utility for the early detection of MAP infected animals. Next, the diagnostic utility of four MAP orthologs (MAP1569, MAP2942c, MAP2609, and MAP1272c) was assessed and reveal moderate to high diagnostic sensitivities (range 48.3% to 76.7%) and specificity (range 96.7% to 100%), with a combined 88.3% sensitivity and 96.7% specificity. Taken together, the results of our analyses have identified several candidate MAP proteins of potential utility for the early detection of MAP infection, as well individual MAP proteins that may serve as the foundation for the next generation of well-defined serological diagnosis of JD in cattle.

## Introduction

Johne’s disease (JD) is a chronic granulomatous intestinal inflammatory disease that results from infection with *Mycobacterium avium* subspecies *paratuberculosis* (MAP) [1]. JD results in more than $200 million in annual losses to the US dairy industry each year [2]. Despite considerable control efforts, JD remains a major problem for producers and the industry due to high prevalence rates (68% of all US dairy herds and 95% of those with over 500 cows have at least one JD positive animal) [3]. Although animals are infected early in life through ingestion of bacilli via the fecal-oral route or from colostrum, JD takes several years to manifest [4, 5]. During this extremely long sub-clinical phase, infected animals are continuously or intermittently shedding the pathogen into the environment and spreading the disease. However, it is very difficult to reliably identify infected from non-infected animals during early infection, especially in animals that are intermittently shedding. Hence, the development of highly sensitive and specific diagnostics has the potential to be transformative in the field and is key for control of JD and enhancement of animal health.

Due to low sensitivity of current serological assays (particularly ELISAs) which use relatively crude cellular extracts, several studies focused on identification of individual antigens soon after the complete genome sequence of MAP was published [6]. These include studies that used bioinformatics’ screens to predict function and localization of proteins, followed by proteomic analyses of cell wall associated proteins [7]; MAP culture filtrates [8]; surface proteins expressed in macrophage [9]; proteins that respond to stress during *in vitro* culture [10]; proteomic comparison of MAP with *Mycobacterium aviam* subspecies *avium* [11]; as well as a dot-blot based protein arrays of recombinant proteins representing secreted or cell wall associated proteins [12] to identify MAP antigens of potential diagnostic utility with varying degrees of success. For instance, studies have shown that sera from experimentally infected cattle recognized specific MAP proteins at a very early stage of the infection, or with either mild or paucibacillary infections that were presumably from subclinical animals and well before antibodies were detected by using commercial ELISA assays [13–15], suggesting that a subset of MAP proteins may be seroreactive during early (subclinical) infection. However, none of these candidates have proved of clinical utility or have shown potential to replace the extant whole-cell antigen based commercially available ELISAs.

To date, more than 200 recombinant proteins have been tested for antigenicity and more than 800 recombinant proteins have been overexpressed for antigen discovery [12–16]. However, this represents only approximately 20% of predicted proteins in the MAP proteome (*n* = 4,350) [6]. Given the significant time and financial costs associated with cloning, expressing and purifying additional proteins from MAP, we have recently explored the possibility of leveraging the commercially available whole proteome microarray from *Mycobacterium tuberculosis* (MTB), a closely related pathogen [17]. The MTB proteome array contains ~4,000 features (3,864 unique MTB genes) covering 97% of the genome and has previously been successfully used to identify biomarkers of active TB infection from a global collection of human and non-human primate serum and plasma samples [18, 19]. Our preliminary pairwise comparison of amino acid sequence between orthologous proteins in MAP and MTB showed an average of 62% identity (range 19% to 100%) with more than half sharing >75% identity [17]. Further bioinformatic analyses confirmed that the MTB proteome array contains ~800 MAP orthologs that have previously been expressed and an additional ~1,900 having significant levels of homology with their MAP orthologs that have not been expressed.

Our pilot studies conducted using serum samples from 9 MAP-infected cows (6 clinical and 3 subclinical) and 3 uninfected control and MTB full-proteome chips revealed more than 700 MTB reactive antigens [17], less than 200 of which represent orthologs that were already represented among the expressed MAP proteins. Probing the MTB array with serum from MAP-infected animals resulted in the identification of more than 500 antigens, for which several of these proteins displayed greater reactivity with serum from subclinical animals as compared to clinical stage animals. This suggests that the MTB protein array has considerable potential to identify a significant number of new candidate antigens detectable during early stages of disease. However, only a very small number of serum samples were used in this preliminary screen, and hence these results needed to be corroborated with an expanded set of well-characterized samples, and further validated for use in immunoassays. We here report immune profiling using a large collection of well-characterized serum samples from MAP-infected cows and negative controls with the MTB protein microarray, as well as the development of specific and sensitive ELISA assays using defined MAP antigens.

## Materials and methods

### Bovine serum samples

All serum samples were collected as part of the Johne’s Disease Integrated Program (JDIP, http://mycobacterialdiseases.org) diagnostic standards sample collection project. In brief, the 180 samples used in these studies were collected from cows housed in 13 dairy farms from 4 states: California, Georgia, Minnesota, and Pennsylvania (S1 Table). The herd size ranged from 66 to 1,400 and prevalence of JD ranged from 0 to 53.30% based on serum ELISA tests conducted prior to sample collection. All herds were negative for bovine TB. As JDIP diagnostic standards sample collection study designed, each cow was tested for level of MAP shedding in feces as well as serological reactivity. MAP shedding was determined by fecal culture using Herrold's solid medium (HEYM) and two different liquid culture medium systems, BACTEC MGIT and Trek (Becton, Dickinson and Company, Franklin Lakes, NJ); all fecal cultures were confirmed by acid fast staining and PCR. Fecal qPCR was performed for each animal with the LT TaqMan (ThermoFisher, Waltham, MA) and Tetracore (Tetracore, Rockville, MD) assays. Serum and milk ELISA tests were performed using both IDEXX kit (IDEXX Laboratories, Inc., ME) and ParaChek (ThermoFisher, Waltham, MA) according to the manufacturers’ instructions. Based on the result of fecal and serological tests, cows were stratified into three groups: both fecal and serological tests negative (*n* = 60), fecal test positive and serological test negative (F+E-, *n* = 60) and both fecal and serological tests positive (F+E+, *n* = 60). Based on the previously observed prevalence of JD in each originating farm (according to serological tests conducted one year before above samples collected), cows in the negative group were further stratified into two groups: negative from low-exposure herds (NL, *n* = 30) if they were from farms that had no recent evidence of JD prevalence (0%) and negative from high-exposure herds (NH, *n* = 30) if the farm had evidence of previous JD prevalence (0.60 to 53.30%).

All serum samples were collected as part of the Johne’s Disease Integrated Program (JDIP, http://mycobacterialdiseases.org) diagnostic standards sample collection project number 2008-55620-18710. Animal use protocols were approved by the Pennsylvania State University IACUC numbers 34625 and 43309.

### Microarray fabrication and probing

The MTB microarray fabrication and probing were conducted in Antigen Discovery Inc. (ADI, Irvine, CA) as described previously [18, 19]. The microarrays carried 3,963 MTB protein spots, which corresponded to more than 97% of the ORFs in the MTB H37Rv genome [18]. Briefly, using genomic DNA as a template, all open reading frames in the MTB H_37_Rv genome were amplified using custom PCR primers. Genes > 3kb in length were amplified as overlapping fragments. PCR products were cloned into a linearized T7 vector using *in vivo* recombination cloning. Using individually purified plasmids, MTB proteins were expressed in an *E*. *coli*-based *in vitro* transcription and translation system (IVTT) (5 Prime, Gaithersburg, MD). The resulting IVTT reactions were printed as single spots without further purification into custom 3-pad nitrocellulose-coated Oncyte Avid slides (Grace Bio-Labs, Bend, OR) using an Omni Grid 100 microarray printer (Digilabs, Inc., Marlborough, MA) in 4x4 sub-array format, with each subarray comprising 18x18 spots. Each sub-array included negative control spots carrying IVTT reactions without DNA templates, purified proteins spots of previously identified MTB biomarkers, as well as positive control spots for the hybridization. Quality control was carried out by probing a sample of chips from each print run using a monoclonal antibody against the N-terminal polyhistidine tag, the C-terminal HA tag and selected reference serum. Cryopreserved serum samples were thawed on ice and pre-incubated with *E*. *coli* lysate to absorb anti-*E*. *coli* and cross-reactive antibodies. Prior to incubation with serum, slides were re-hydrated and blocked for 30 minutes using Blocking Buffer (Main Manufacturing, Sanford, ME). Serum samples were diluted 1:200 and incubated on arrays at 4°C overnight with gentle agitation. Bound IgG antibodies were detected with a biotinylated anti-bovine IgG secondary antibody (Jackson ImmunoResearch, West Grove, PA), followed by incubation with Surelight-P3 fluorochrome conjugated to streptavidin (Columbia Biosciences, Columbia, NY). Slides were then dried and scanned in a Genepix 4300A microarray scanner (Molecular Devices, San Diego, CA). The scanner laser power and PMT gain were calibrated daily to intensities obtained from reference sera to control for day-to-day variation. Fluorescence intensity values for each spot were quantified using GenePix Pro software, and data were exported in comma separated values (CSV) format (intensity data accessible via https://scholarsphere.psu.edu/concern/generic_works/hhm50ts37m).

### Data analysis

The intensity data files in CSV format were read, processed and analyzed using an automated data analysis pipeline developed at ADI that was implemented in R (www.r-project.org). Spot intensity measurements were converted into a single data matrix of local background-subtracted intensities. The row names of the data matrix are unique spot identifiers that link to a spot annotation database, and the column names are unique sample identifiers that link to a sample information database. For each sample, quality checks were performed for possible missing spots, contaminations and unusual background variation. The data were also inspected for the presence of subtle systematic effects and biases (probing day, slide, pad, print order, etc). Once the data passed quality assurance, the final dataset utilized for analysis was obtained by the following steps: (1) log_2_ transformation of raw intensities; (2) for each sample, calculation of the median of the IVTT negative control spots; and finally (3) subtraction of the sample-specific IVTT negative control medians. An antigen is classified as highly reactive to a given sample if its normalized intensity value is greater than 0.5 (the raw intensity is at least approximately1.4x the sample’s median IVTT negative control). An individual’s antibody breadth scores are determined by its count of reactive antigens. Antibody breadth profiles were compared between groups using Poisson regression. Normalized data were modeled using parametric and non-parametric tests for between-group comparisons. For complex data sets, comparisons were made using multivariate linear regression or linear mixed models with random effects for longitudinal data. All *p*-values were adjusted for the false discovery rate as previously described [20].

### ELISA assay for selected MAP recombinant proteins

ELISA assays were conducted for selected MAP recombinant proteins (their MTB orthologs were identified as significantly reactive antigens) with serum samples from NL and F+E+ groups. The procedure was adapted from our previously described protocol [17] with a minor modification. ELISA 96-well microplates were coated with 50 μl/well of 1 μg/ml recombinant MAP protein or 0.5 μg/ml MBP/LacZ (fusion protein from cloning vector) in carbonate/bicarbonate buffer 0.1 M pH 9.6. Plates were sealed and incubated overnight at 4°C, then washed three times with 1xPBS, pH 7.4 containing 0.1% Tween 20 (PBS-T). Wells were blocked by adding 200 μl/well of PBS-T containing 1% bovine serum albumin (PBS-T-BSA) and incubated at room temperature for 1 hour before washing the plate three times with PBS-T. Serum samples diluted 1:250 in PBS-T-BSA were added to each well (100 μl/well) and incubated at room temperature for 1 hour before washing six times with PBS-T. Then 100 μl/well of anti-goat IgG peroxidase conjugate (Vector Labs, Buringame, CA, USA) diluted 1:10,000 in PBS-T-BSA was added to all wells and incubated at room temperature for 1 hour before the plates were again washed six times with PBS-T. Finally, 100 μl/well of tetra methylbenzidine (TMB) SureBlue solution (KPL, Gaithersburg, MD, USA) was added and the reaction incubated for 10–15 minutes at room temperature with no light, before the reaction was stopped with 100 μl/well of 1.0 N HCl solution. The spectrophotometric reading of all wells was performed at 450 nm using a PowerWave XS2 microplate reader (BIoTek, Winooski, VT, USA). The OD value of each sample was normalized by sample OD–MBP/LacZ OD to eliminate the non-specific background produced by anti-MBP/LacZ in each serum sample. The group *t* test was performed using GraphPad software (https://www.graphpad.com) and the significance of correlation of coefficient was determined using an online statistical computation tool (http://vassarstats.net/index.html).

### Logistic regression analysis

To determine which antigens had significantly different normalized intensities values among the 4 groups (NL, NH, F+E-, F+E+), ordinal logistic regression models were fitted, using PROC LOGISTIC in SAS (version 9.2, 2009; SAS Institute Inc., Cary, NC). Such models are appropriate for outcomes with more than two categories, as in this study, where the outcome was group with 4 categories (NL, NH, F+E-, F+E+). Each antigen was included in a model one at a time; all models also included lactation number of the cow, day-in-milk, and herd size. In each model, the generalized logit function was specified; each nonbaseline category is compared to the baseline category. In each model run, 180 observations were read in, but only 167 were used in the analysis, due to missing values for some covariates. Statistical significance was considered at alpha = 0.05.

The output produced was in the form of odds ratios and their 95% confidence limits, for each category of group within a covariate (antigen, lactation number, day-in-milk, herd size). The baseline category varied with model, as it was desirable to have the baseline odds ratio value for each antigen be 1.0, and all comparisons made to that, within each antigen of interest, such that all comparison values were greater than 1.0. Therefore, each comparison (odds ratio for a particular group) gave the odds of belonging to a particular group compared to the odds of belonging to the baseline group. The odds ratio indicates how likely a certain antigen is associated with a particular group, compared to being associated with the baseline group. Another way to view the findings is thus: if, for a particular antigen, the odds ratio for NL is 1.0 (baseline group) and the odds ratio for F+E+ is 2.5, then for each unit increase in the normalized intensity value of the antigen, a cow is 2.5 times more likely to be classified as F+E+ than as NL.

## Results

### Identification of highly reactive proteins

**Fig 1:**
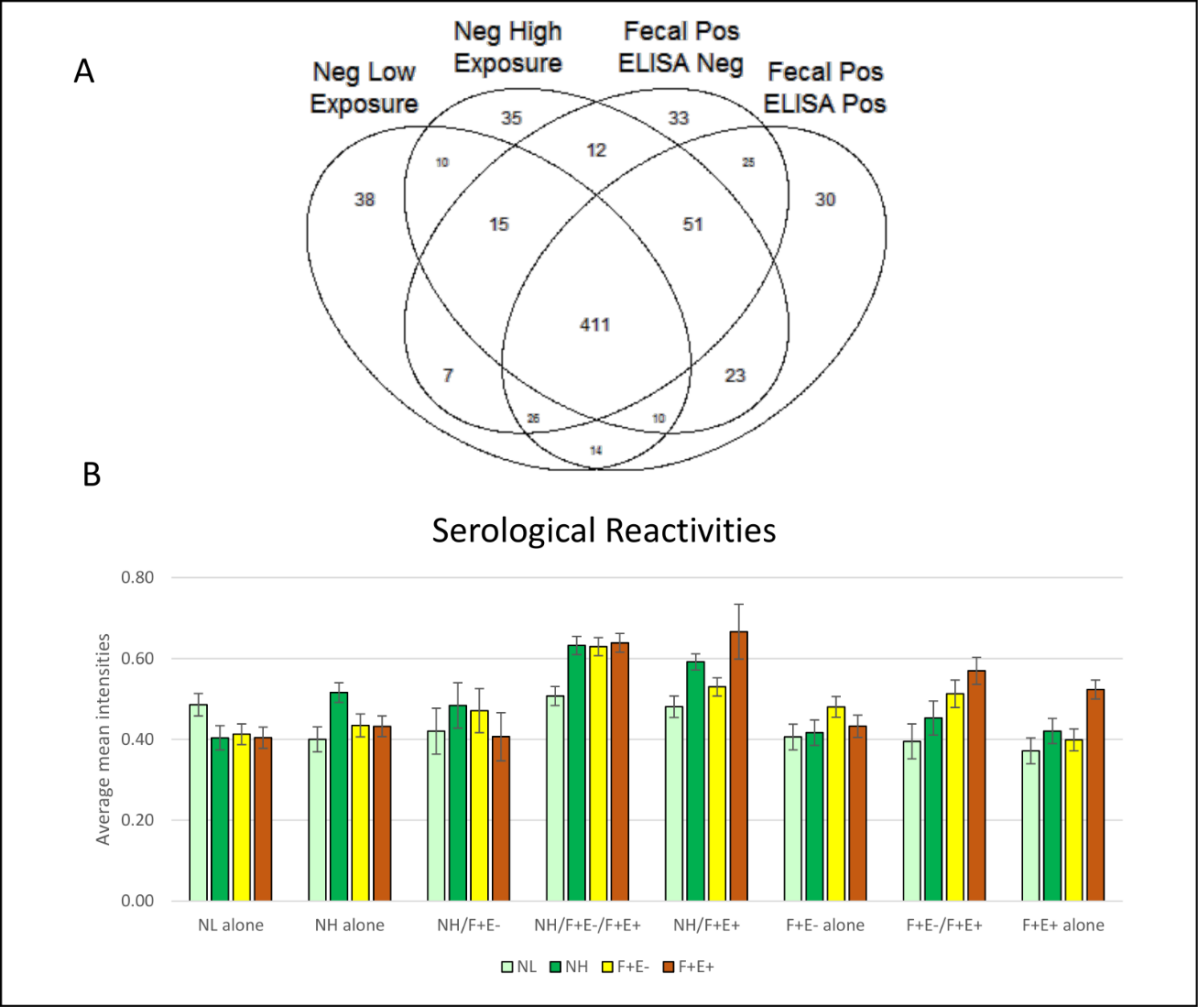
Highly reactive proteins identified in MTB microarray. (A) Venn diagram at 10% threshold shows antigen hit number distribution of all 4 groups: negative low exposure (NL), negative high exposure (NH), fecal positive & ELISA negative (F+E-), and fecal positive & ELISA positive (F+E+). The four ellipses show the total number of hits from four groups and majority antigens are shared among 4 groups. The non-overlapping parts of the 4 ellipses represent the unique antigens for each group. (B) Serological reactivity in selected groups. Normalized mean intensities in each group on unique and shared antigens. The height of bars represents the mean intensity. Standard error bars are added to each column.

A total of 740 highly reactive antigens were identified based on normalized intensities at a 10% threshold with a distribution amongst the NL, NH, F+E-, and F+E+ groups as shown in the Venn diagram (Fig 1A). In brief, the four ellipses show the total number of hits from the four groups of animals, with the majority of reactive proteins sharing cross-reactivity. If a highly reactive protein was identified in one group only, the protein was categorized as a unique protein. If a reactive protein was identified in two or more groups, the protein was categorized as a shared protein. Proteins were divided into 15 categories based on their unique or shared status among the groups. Unique proteins were identified in each of the 4 groups as: 38 in NL (5.1%), 35 in NH (4.7%), 33 in F+E- (4.5%) and 30 in F+E+ (4.1%) group respectively. There were a total of 411 proteins shared among all 4 groups, accounting for 55.5% of the total reactive proteins identified. The remaining proteins were shared within two (12.3%) or three (13.8%) of the groups. The average normalized intensities of proteins shared by all 4 groups were highest (> 1.0), while the average intensities of the other groups were between 0.37 and 0.67 (Fig 1B).

### Identification of significantly reactive proteins

To determine which of the two groups of negative samples should be used as reference for group comparisons (NL or NH), we compared the mean intensities of the infected groups (F+E- and F+E+) with that of NL and NH individually as a reference. When mean intensities of the NL group were used as reference, 39 and 76 proteins were identified as significantly reactive proteins (P <0.05, based on group *t* test) in the F+E- and F+E+ groups, respectively. However, when the mean intensities of the NH group were used as reference, the number of significantly reactive proteins was reduced to 12 and 26 in the F+E- and F+E+ groups, respectively (S1A and S1B Fig). There were only 5 proteins shared in F+E- and 15 in F+E+ groups when mean intensities of NL and NH were used as a reference, respectively. In light of these observations, we chose to use NL alone as a reference for two reasons: 1) antigen identification was very reference-dependent and 2) samples from animals early in infection may contain antibodies recognizing MTB antigens in NH, and therefore candidate antigens may not be recognized if the mean intensities of NH are used as a reference. Mean normalized intensities in each group were compared to NL with a two-tailed *t*-test using a *p*-value < 0.05 for significance. Of the 740 highly reactive proteins from the MTB array, approximately 13% were identified as significant (100 proteins) using this test. Among the 100 identified MTB proteins, there were a total of 69 unique proteins in the groups (9 in NH, 13 in F+E-, and 47 in F+E+) and 31 shared among groups (S2 Fig). On the other hand, if the mean intensities of proteins were significantly higher in the NL-alone group or groups shared with NL compared to the other three groups, these proteins were not considered as significant antigens, or “hits” (Fig 1B). Significant antigens were identified in the following groups (number of significant antigens/total in group; percentage of significant antigens in the group): NH alone (5/35, 13.8%), NH/F+E- (1/12, 8.3%), NH/ F+E+ (8/23, 34.8%), NH/F+E-/F+E+ (21/51, 41.2%), F+E- alone (6/33, 18.2%), F+E-/F+E+ (10/25, 40.0%) and F+E+ alone (11/30, 36.7%).

### Patterns of intensity changes among three groups

**Fig 2:**
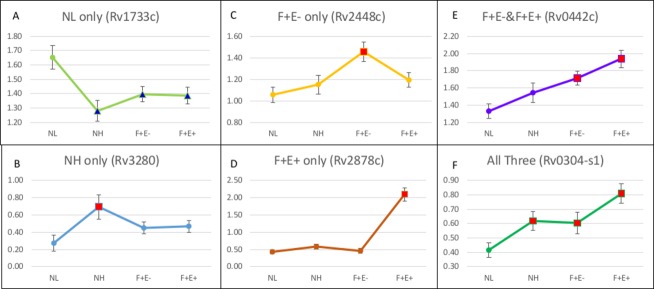
Patterns of mean intensities in each group. The red square markers represent mean intensities significantly higher than that in NL and blue triangle markers indicate mean intensities significantly lower than that of NL. Standard error bars are added to each spot. Mean intensities are significantly higher: A. in NL; B. in NH; C. in F+E-; D. in F+E+; E. in F+E- and F+E+; F. in all three groups including NH, F+E-, and F+E+ (Y axis: mean intensity of each group).

Compared to the normalized mean intensity of each protein in NL, there were 27 proteins with significantly higher and 15 with significantly lower intensities identified in the NH group (P < 0.05). For the majority of proteins, the trend of intensity changes in the NH group was consistent with the changes in infected groups. For example, up to two thirds of proteins identified in NH were also found to have significantly higher (or lower) intensities in F+E- or F+E+ or both groups (S2 Fig) when compared with NL. Similar to NH, two thirds of the proteins identified in F+E- group were also shared with other groups, while in F+E+ group, more than 60% of proteins were unique. There were 6 patterns of intensity changes among three groups in comparison with NL (Fig 2). The first pattern shows mean intensities are significantly higher only in NL (Fig 2A). Among the 15 proteins with significantly lower intensities in NH, 14 were also found with lower intensities in F+E- and F+E+ groups. Only one protein, Rv0040c (Fig 2E; ortholog MAP0047c), showed significant lower intensities in NH and F+E-, but significantly higher intensities in F+E+. Compared to intensities in NL, the proteins with lower intensities in infected groups were not considered reactive antigens, while proteins with significantly higher intensities in the other three groups were considered reactive antigens following the described 5 patterns. Proteins with significantly higher intensities only in NH group (Fig 2B) were considered to be antigens recognized only during the early stage of infection. Proteins with significantly higher intensities only in F+E- (Fig 2C) or only in F+E+ (Fig 2D) indicate that the antigen is recognized only in the middle or late stages of infection, while proteins with significantly higher intensities in both F+E- and F+E+ groups (Fig 2E) or in all three groups including NH, F+E- and F+E+ (Fig 2F), indicate antigens that can be recognized throughout the course of infection.

### Orthologs in MAP

**Fig 3:**
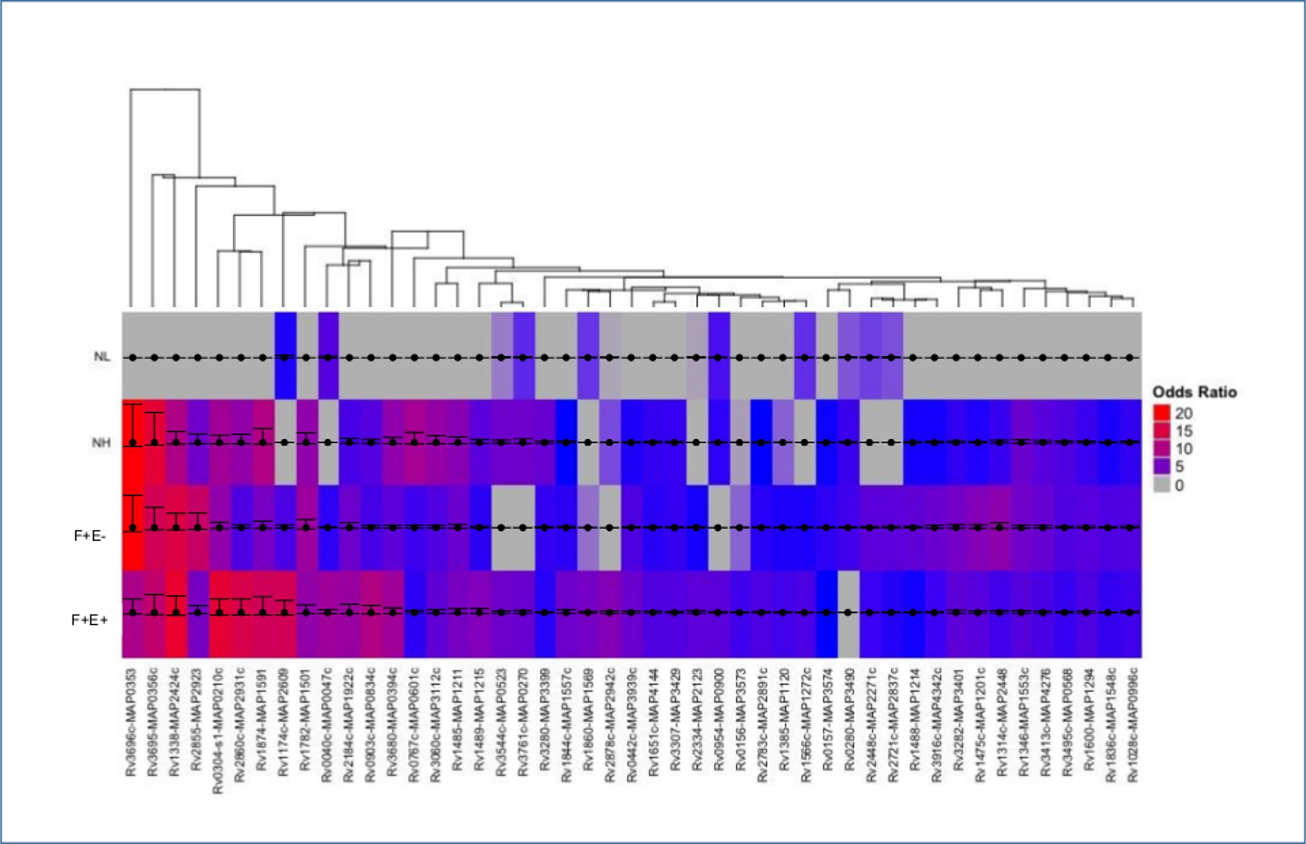
Patterns of serum reactivity to MTB proteins with their odds ratios that differed significantly in at least one of the 4 groups. The heatmap shows the odds ratio of the serum reactivity from 4 groups to each of the 47 proteins. Each column represents one protein, odds ratios (rows) are visualized as a color spectrum. The heatmap was generated using the ComplexHeatmap package in R. The clustering was performed using the pvclust package with multiscale bootstrap resampling. Arguments passed to the pvclust command for the hierarchical clustering method (method.hclust) was “median” and for the distance method (method.dist) was “maximum”. The confidence intervals were overlaid on the heatmap using the ggplot2 package.

Among the 100 significantly reactive MTB proteins, there were 91 proteins with mean intensities close to or higher than 0.5 and 9 proteins with intensities lower than 0.5. Normalized intensities at 0.5 indicated an approximately 41% higher signal than background where 0 represents the equivalence with background intensities. Among these 9 proteins, mean intensities in the NL group were near 0 and mean intensities in infected groups were more likely to be significantly higher even mean intensities are slightly increased when compared to NL. Therefore, these 9 proteins were excluded to avoid false positives. For the remaining 91 proteins identified in the MTB array, the MAP orthologs were determined based on the comparison of their amino acid sequences and the patterns of antigenicity between the MTB protein identified on the array and the corresponding MAP ortholog. Specifically, for a MAP protein to be considered an ortholog of the identified MTB protein, the amino acid sequence identity must be ≥ 40%. However, some proteins, such as Rv0304c-s1 and MAP0210c, which have an overall low identity but show a higher identity in the antigenic regions, are also considered to be MAP orthologs. While the majority of MTB proteins match one single MAP protein, in some cases there are two or more MTB proteins matching the same MAP ortholog, such as Rv0304c & Rv1004c to MAP0210c; Rv1677 & Rv2878c to MAP2942c; Rv1651c & Rv2328 to MAP4144. MAP orthologs were selected from the infected groups based on percent sequence identity and mean intensity values of corresponding MTB proteins on microarrays. For instance, 5 MTB proteins (Rv1753c, Rv0442c, Rv1918c, Rv1917c, and Rv3350c) match MAP3939c with identities ranging from 58.2% to 72.2% at the amino acid level (S3A Fig). These 5 MTB orthologs are PPE family proteins with an identity between 49% and 71% between each other. However, Rv0442c is the most closely related ortholog with an amino acid sequence identity of 72.2% and the highest mean intensity. The MAP3939c and Rv0442c also showed similar antigenicity patterns (S3 Fig and Fig 3B). A total of 73 MAP orthologs were determined from initial 100 significant MTB antigens identified from MTB array. The logistic regression analysis was applied to 73 MTB ortholgs and ordinal logical regression models were fit. In each model the baseline has an odds ratio of 1.0, and all the other categories have odds ratios greater than 1.0, compared to the baseline. Among 73 proteins, there are 47 proteins having significantly different normalized intensity values in at least one group (p<0.05). The remaining 26 antigens did not significantly differ in any of the 4 groups and were excluded as antigens. The 47 antigens were visualized in the heatmap showing the odds ratios for serum reactivity to each antigen among 4 groups (Fig 3). S2 Table listed all 47 MAP proteins with the identity to their MAP orthologs, predicted cell location, functional description, and odds ratios among 4 groups (S2 Table).

### Recognition of identified reactive antigens in previous studies

Several MAP orthologs that were identified in the MTB microarray were also recognized in previous studies by other researchers. For instance, the orthologs MAP2609, MAP2942, and MAP0210c were previously characterized as secreted 9, 15, and 34 kDa MAP antigens, which were recognized by antibodies from naturally infected cattle at both clinical and subclinical stages [21]. The ortholog MAP1569 (ModD) was also identified as a secreted protein that was recognized by sera collected from naturally infected cows [22, 23]. The ortholog MAP0834c, a two component system transcriptional regulator, was recognized by sera from naturally MAP infected sheep as a significantly reactive antigen [24]. Another ortholog MAP1272c, an invasion-associated protein, has been identified in several studies as one a promising antigen [24, 25] and recently further characterized on crystal structures, combined with functional assays [26]. The ortholog MAP0900 (P35), a conserved membrane protein, was recognized by 100% of animals including cattle, goats and sheep with Johne’s disease in the clinical stage and 75% of cattle in the sub-clinical stage [27], as well as 75% of patients with Crohn’s disease [28]. One protein, Rv1411c (ortholog MAP1138c), significantly reactive in F+E+ group but not listed as identified MAP orthologs due to low mean intensities (<0.5), was also recognized in previous studies as immunogenic [29]. Antibody to expressed recombinant protein MAP1138c (P22) was detected in sheep vaccinated by a MAP strain and also in clinical/subclinical cows with Johne’s disease [29]. The recombinant P22 (MAP1138c) was able to stimulate significant IFN-γ production in blood of P22-immunized sheep [30]. It needs to be noted that all of the above proteins in previous studies were tested in a relatively small number of infected animals and the majority of animals were tested positive with commercially available ELISA tests. About 90% of identified orthologs with the MTB microarray assays in this study have never been tested for their serological reactivity on a large scale set of serum samples.

### Sensitivity and specificity of identified top antigens

**Fig 4:**
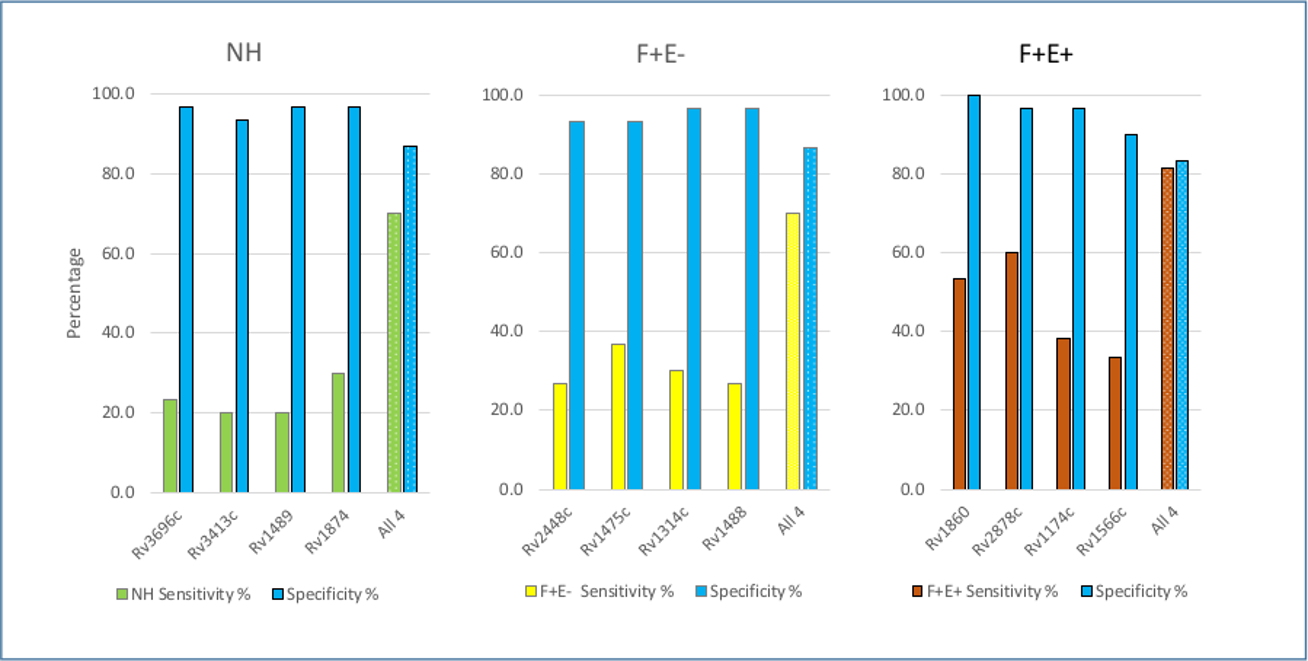
Increased sensitivities with antigen combinations. Columns filled with blue color represent specificity (%) and columns filled with green, yellow, and crimson represent sensitivity (%) in NH, F+E-, and F+E+ groups. Columns filled with solid color indicate individual protein and columns filled with texture indicate combined proteins.

Our goal was to establish a collection of antigens that could be used as a multiplex set to accurately distinguish MAP-infected animals from non-infected animals. To do this, we compared the sensitivity and specificity for each of the 73 identified proteins at both mean + 1 standard deviation (1SD) and mean + 2SD level (S3 Table). Specificity at the M+1SD cutoff is between 63.3% and 93.3% with a median of 83.3%, and increased to 73.3% to 100.0% with a median of 96.7% at the M+2SD cutoff. Sensitivities for the majority of single proteins were low with median sensitivities of 33.3%, 28.3%, and 30.5% at M+1SD cutoff in NH, F+E-, and F+E+ groups, respectively, and further reduced to 16.7%, 16.7%, and 15.0% at the M+2SD cutoff. Based on comparison of odds ratio and sensitivity/specificity for each protein, we focused on proteins with relatively high sensitivity/specificity and compared different combinations of several proteins to find the best combination with high sensitivity without significantly lowering specificity. For each of group NH, F+E-, and F+E+, we selected a combination of 4 proteins. At the M+1SD cutoff, the sensitivity with the 4 combined proteins significantly increased and reached 80.0%, 85.0%, and 88.3% in the NH, F+E-, and F+E+ groups respectively, however, the specificity dropped from above 90.0% with a single protein to 43.3% and 73.3%, respectively. To avoid false positives, we chose a cutoff at M+2SD level and the sensitivity at each group significantly increased with specificities all above 80.0% (Fig 4). These results indicate that using a combination of antigens greatly increases the sensitivity in detecting MAP with only a relatively small reduction in specificity.

### Reactivity of MAP orthologs confirmed on ELISA

**Fig 5:**
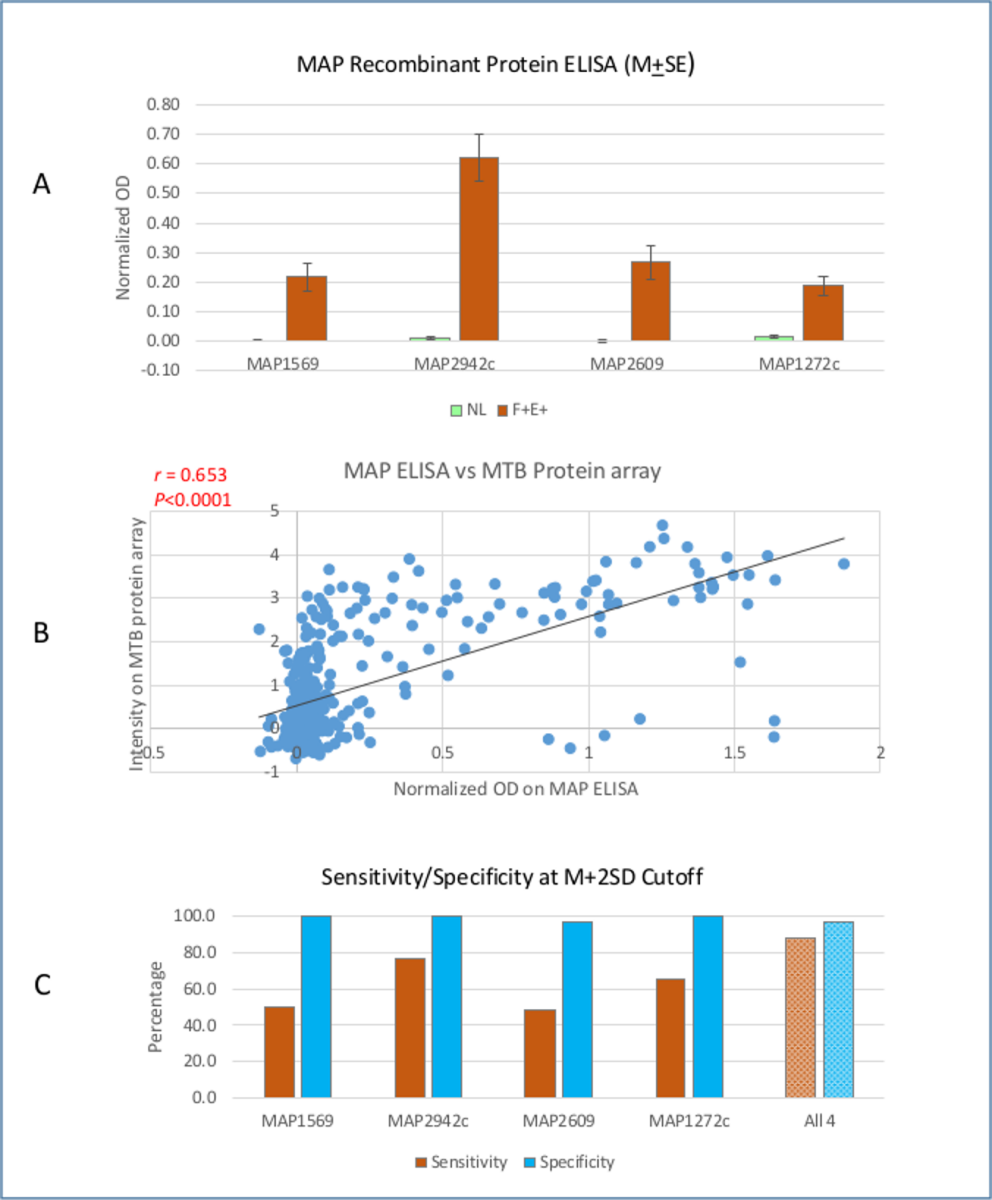
Reactivity of MAP orthologs on ELISA. A. Group comparison between NL and F+E+ in selected MAP recombinant protein ELISA. B. Correlation between MAP ELISA and MTB protein array. C. Sensitivity and specificity with individual MAP recombinant protein and combined 4 proteins at M+2SD cutoff.

To evaluate if antigens identified with the MTB protein microarray are reactive in infected cows, four recombinant proteins of MAP orthologs (MAP1569, MAP2942c, MAP2609, and MAP1272c corresponding to Rv1860, Rv2878c, Rv1174c and Rv1566c) were selected for ELISA with 90 serum samples including 30 from NL and 60 from F+E+. The identities of these four orthologs between MAP and MTB are from 61.8% to 77.6%. The normalized OD values in two groups were compared and OD values in F+E+ group were significantly higher than that in NL group with p < 0.01 for all 4 antigens (Fig 5A). This result was consistent with the group comparison in MTB protein array, but the background was much lower in NL group, and the ratio of positive/negative was greatly increased in the MAP ELISA. Correlation between the seroreactivity of antigens on the MAP ELISA and orthologs on the MTB array was also examined. For each serum sample, the normalized OD on MAP ELISA was compared to intensity on MTB array and the correlation coefficient, Pearson’s rho, was from 0.395 to 0.796 with the lowest in MAP1569 and the highest in MAP2942c (p value < 0.0001 in each of the antigens). Fig 5B showed correlation among all 4 proteins (rho = 0.653, p < 0.0000001), indicating strong correlation between serological reactivity of infected cows to MTB antigen and MAP orthologs. These data suggest that MTB orthologs on the MTB arrays react to serum from MAP-infected cows in a manner similar to MAP ELISA with MAP recombinant proteins. Based on ELISA data, the sensitivity and specificity for detection of infection was examined and compared with that in MTB protein array at M+1SD and M+2SD cutoff levels. At M+1SD cutoff, the sensitivity on each individual antigen ranged from 55.0% to 81.7% with specificity 83.3% to 96.7%. With 4 antigens combined, the sensitivity increased to 96.7%, but specificity was reduced to 70.0%. At M+2SD cutoff, although sensitivity of each individual antigen was reduced (48.3% - 76.7%), the specificity ranged from 96.7% to 100%. With 4 antigens combined, sensitivity increased to 88.3% with specificity 96.7%. Compared to the MTB array on these 4 antigens, MAP ELISA displayed higher sensitivity and specificity. The consistency of group comparison and strong correlation between MTB array and MAP ELISA indicate that antigenic orthologs identified on MTB protein array with serum samples from cows are capable of distinguishing infected cows from uninfected cows.

## Discussion

Generally, determination of significantly reactive antigens for recombinant proteins is based on the comparison of serological reactivity of infected animals to uninfected animals. Usually, when an animal tests MAP negative for both fecal (culture or PCR) and ELISA (serum or milk), we consider the animal to be not infected. However, in this case, the uninfected status may not be true because MAP infection at the tissue level is unknown. Several studies have shown that cattle determined not to be shedding based on either fecal culture or PCR were later found to be MAP-infected in their tissues at the slaughterhouse. Whitlock *et al*. reported that more than 30% of fecal culture negative cattle from moderately infected herds (fecal culture positive ranging between 5% and 15%) have infected tissues taken at the time of slaughter [31]. Another study comparing MAP culture and PCR in fecal and tissue samples from intestine and the mesenteric lymph node found that MAP was detected by PCR and isolated from tissues in some cattle testing fecal negative [32]. A recent study compared the lymphatic fluid, fecal material, and antibodies from serum and milk samples (ELISA) for detection of MAP infection in cows. The results showed that more than two thirds of animals with a positive lymph result were negative in all fecal and ELISA tests and only 7% of the animals with positive lymph-PCR were also positive in all other tests [33]. Taken together, these results indicate that some animals with negative fecal and ELISA tests are not a true negative.

In this study, 60 samples with both fecal and ELISA negative results were divided into two groups, NL and NH, according to the prevalence of the farms where the samples were collected. By comparing the means of normalized intensities between these two groups, we identified 27 proteins with significantly higher reactivity. Among the 27 identified proteins, two thirds were also shared with F+E-, F+E+, or both, indicating the proteins identified in NH are likely to be true antigens. We hypothesized that cows in the NH group may not be true negatives and were probably in early stage of infection. We found that if NH was used for reference, only 31% and 34% of reactive antigens were identified in the F+E- and F+E+ groups respectively, as compared when NL was used as a reference. Because it is important to select true negatives as a reference to identify reactive antigens in the infected groups of animals we analyzed our data set using NL as the reference.

We hypothesized the stages of infection in the cows as follows; NL = Uninfected; NH = Early; F+E- = Middle; and F+E+ = Late stage of infection. There is no significant difference in average lactation number among the 4 groups: NL is 3.13 (SD = ±1.46), NH 2.93 (SD = ±1.08), F+E- 2.95 (SD = ±1.06), F+E+ 3.32 (SD = ±1.40) (S1 Table). All infected cows are likely to be in the sub-clinical stage because there were no clinical signs of Johne’s disease recorded. As mentioned above, NH showed a different profile of serological reactivity to recombinant proteins compared to NL despite the negative results from the fecal exam and commercial ELISA. Therefore, we speculated that cows in NH were infected with MAP at the early stage. At this stage, serological reaction with traditional commercial ELISA is unlikely to be detected according to experiments in cows with established MAP infection. The time required for seroconversion in experimentally infected calves detectable by commercially available ELISAs is between 10 and 28 months [34]; and it may take possibly longer in naturally infected animals. Although animals generally shed MAP in their feces before seroconversion, the chance of detecting MAP shedding at this stage is very low due to intermittent shedding as observed in many experimentally infected animals [35]. A comparative investigation on cows in slaughterhouses demonstrated viable MAP (or MAP DNA) isolated from mesenteric lymph nodes and intestinal tissues but not from feces in some cows [32], indicating that negative fecal tests could not exclude infection in gut tissue. The other two infected groups, F+E- and F+E+, were both positive in fecal testing, with or without positive ELISA, but the bacterial burden in feces was significantly different (P<0.001). According to two fecal qPCR tests, the average Ct values in F+E- were 35.6 (SD = ±2.7) and 37.7 (SD = ±2.5), compared to 26.7 (SD = ±4.1) and 29.8 (SD = ±4.2) in F+E+, indicating that the MAP burden in the F+E+ group was at least 100 times higher than the F+E- group. The cows in F+E- were considered to be low shedders while the F+E+ group contained high shedders. Based on the quantity of fecal MAP shedding and serological reactivity (ELISA) results, it is reasonable to assign cows in the F+E- group as middle stage infection and the F+E+ group as late stage infection. In previous studies, cows have usually been classified as negative, sub-clinical, and clinical. In this study, we further divided sub-clinical into early, middle, and late stages and identified unique and shared reactive antigens at these different stages of infection.

Currently available ELISA methods are not able to detect serological reactivity during early infection, as shown previously and confirmed in this study and ELISA results only appear as positive during the later stages of infection. With the completion of the genome sequence of MAP K10, it became possible to identify potentially antigenic proteins at a full proteome scale [6], and follow-up studies focusing on the ontogeny of the humoral response to MAP led to identification of antigens marking the early stages of infection. For instance, in experimentally infected cattle, some recombinant MAP proteins were identified on the basis of the humoral immune response as early as 70 days after infection [36]. These identified antigens were also recognized by sera from naturally infected cattle in the sub-clinical stage of Johne’s disease. Other studies with MAP experimentally infected cattle showed that the antibody against the recombinant protein (MAP1197) was detected 2–7 months earlier than a commercially available ELISA kit and even earlier than shedding in some cattle [14]. In naturally infected sheep with mild histological lesions of paratuberculosis, more than half of the serum samples had detectable antibody responses against recombinant MAP proteins, but no response to the commercial ELISA [13]. Although promising, a comprehensive identification of the most promising antigens during early stages of MAP infection was limited by several factors. First, there was no well-characterized collection of serum samples from naturally infected animals available to validate recombinant proteins and naturally infected host animals since these were often not classified by different stages of sub-clinical infection. Second, it is difficult to screen large numbers of recombinant proteins using standard ELISA or western blotting techniques, as performed in previous studies. To overcome these limitations, during this investigation, we used a total 180 serum samples from well characterized animals for screening of ~4,000 recombinant MTB proteins and identified reactive antigens at stages of early, middle, and late infection. A total of 12 and 23 MAP orthologs were identified in the NH and F+E- groups, respectively, although all cows in these two groups showed negative serological reaction based on commercial ELISA tests on both serum and milk samples. Fifty-three MAP orthologs were identified from F+E+. We compared the sensitivity and specificity of each identified ortholog and tested if the sensitivity increased without losing specificity. As a result, 4 proteins were selected from each group and combining these 4 antigens increased sensitivity without an appreciable loss in specificity. As shown in Fig 4, sensitivity increased from 20.0–30.0% with a single antigen to 60.0% with the 4 combined in NH, 26.7–36.7% to 63.3% in F+E-, and 33.0–60.0% to 81.7% in F+E+. Compared to results with commercial ELISA methods, there is considerable advantage for detection of reactive antigens with recombinant proteins during the early and middle stages of infection because there was no detectable antibody response against a crude mixture of antigens with commercial methods. However, at the late stage of infection (F+E+) with high shedding levels, commercial ELISA methods showed higher sensitivity as compared to recombinant proteins. This is consistent with previous studies showing that ELISA has a higher sensitivity in animals with a heavier bacterial load (high shedders) compared to low shedders [31]. Combined recombinant proteins showed increased sensitivity for detection of infected cows in this study and we will plan to test more combination of different proteins to improve the detection of infected animals in the future study.

While eight of the significantly reactive antigens identified with the MTB protein array in our current investigation have also previously been reported to be recognized in sera from animals with subclinical and clinical infection [21–27, 29], a majority of the others have not, suggesting that the protein microarray approach has considerably utility for diagnostic antigen discovery. Further, our analyses suggest that the serological reactivity to MAP recombinant proteins with ELISA is consistent with reactivity to MTB ortholgs on MTB arrays with a strong correlation between reactivity to MTB orthologs on the protein array and to MAP proteins on ELISA. These results are consistent with our earlier finding of concordance in scale and direction of serological reactivity between MTB and MAP arrays [17].

A majority of MAP proteins that were previously described as “non-antigenic” were also not reactive in the MTB array, having either very low mean intensities or no significant difference between the infected and control groups. On the other hand, some of the proteins previously recognized as sero-reactive failed to be recognized as significantly reactive on the MTB arrays. This could be due to the fact that: (i) the previously recognized MAP proteins had no homologs in MTB; (ii) identity of orthologs is too low for a MTB spot to be recognized by antibodies against MAP orthologs; (iii) since there was only a small number of samples tested in most of the previous studies, the results may not accurately reflect the true status; or (iv) some antigens may have been identified in experimentally infected animals and there might be differences in serological response between natural and experimentally infected animals. The utility of the MTB array is limited when MAP proteins are either not represented or have low levels of similarity to their MTB orthologs. For example, MAP2121c, a 35 kDa major membrane protein (MMP) was identified as a reactive antigen in several MAP studies [36–39], has no ortholog in MTB. Similarly, a cluster of MAP proteins from MAP0851-0865 have no orthologs in MTB and are thus not included on the array even though several proteins in the cluster were identified as antigenic in previous studies [12, 40].

It is important to note that all 8 proteins identified both in this MTB array study and previous studies were only found in the F+E+ except for one (MAP0210c, Rv0304), which was also recognized in cows from the NH and F+E- groups. This is probably because the majority of infected animals used in previous studies were at clinical or late sub-clinical stages, and the majority of cows in this study (such as NH and F+E- groups) were at early or middle stages of infection. About 80% of identified orthologs with the MTB microarray in this study have never been tested in previous studies for their serological reactivity with a robust and representative serum bank, and many of these candidates will need to be expressed and added to the MAP protein array for future studies.

In conclusion, the results of our studies have led to the identification of a large number of promising candidates antigens that provide a strong framework for the future development of the next generation of highly sensitive and specific diagnostic assays for the diagnosis of early MAP infection in cattle and other susceptible hosts.

## Supporting information

S1 Table Serum samples used in the study. (XLSX)

S2 Table MAP orthologs of MTB proteins identified as significantly reactive antigens. (XLSX)

S3 Table Sensitivity and specificity for each antigen. (XLSX)

S1 Fig Different profiles of comparison of infected groups with NL and NH as a reference. A. Number of significantly reactive proteins identified in F+E- group in comparison with NL and NH. The large circle represents the number of significantly reactive proteins in comparison with NL and the smaller circle represents the number of identified proteins in comparison with NH. The overlap part represents the number of proteins shared. B. Number of significantly reactive proteins identified in F+E+ group in comparison with NL and NH.
(TIFF)

S2 Fig Proteins identified in NH, F+E-, and F+E+ group. Unique proteins represents proteins the proteins are significantly reactive (P<0.05) proteins identified only in the specific group (NH, F+E-, or F+E+). Shared proteins represent proteins are significantly reactive (P<0.05) proteins identified in two or three groups.
(TIFF)

S3 Fig Comparison of MAP3939c with 5 MTB orthologues. Upper: multiple alignment of MAP3939c with 5 MTB orthologue. As showed in the alignment, there is the highest identity between MAP3939c and Rv0442c. Bottom: similar structure characters between MAP3939c and Rv0442c (Protean of Lasergene, DNAstar, Madison, Wisconsin).
(TIFF)
