# Supplementary figures and images for: Identification of sero-reactive antigens for the early diagnosis of Johne’s disease in cattle
Source: PLoS One. 2017 Sep 1;12(9):e0184373. doi: 10.1371/journal.pone.0184373 (PMC5581170; doi:10.1371/journal.pone.0184373)

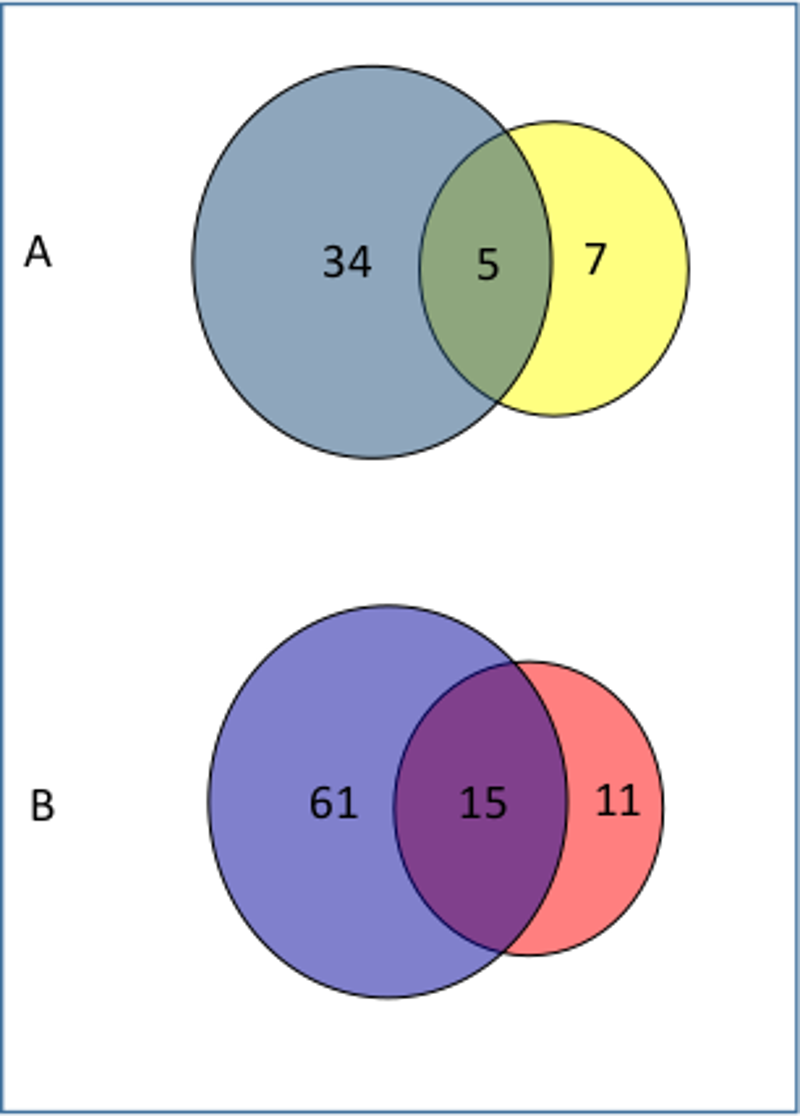

Supplement: S1 Fig — A. Number of significantly reactive proteins identified in F+E- group in comparison with NL and NH. The large circle represents the number of significantly reactive proteins in comparison with NL and the smaller circle represents the number of identified proteins in comparison with NH. The overlap part represents the number of proteins shared. B. Number of significantly reactive proteins identified in F+E+ group in comparison with NL and NH. (TIFF) [file pone.0184373.s004.tiff]

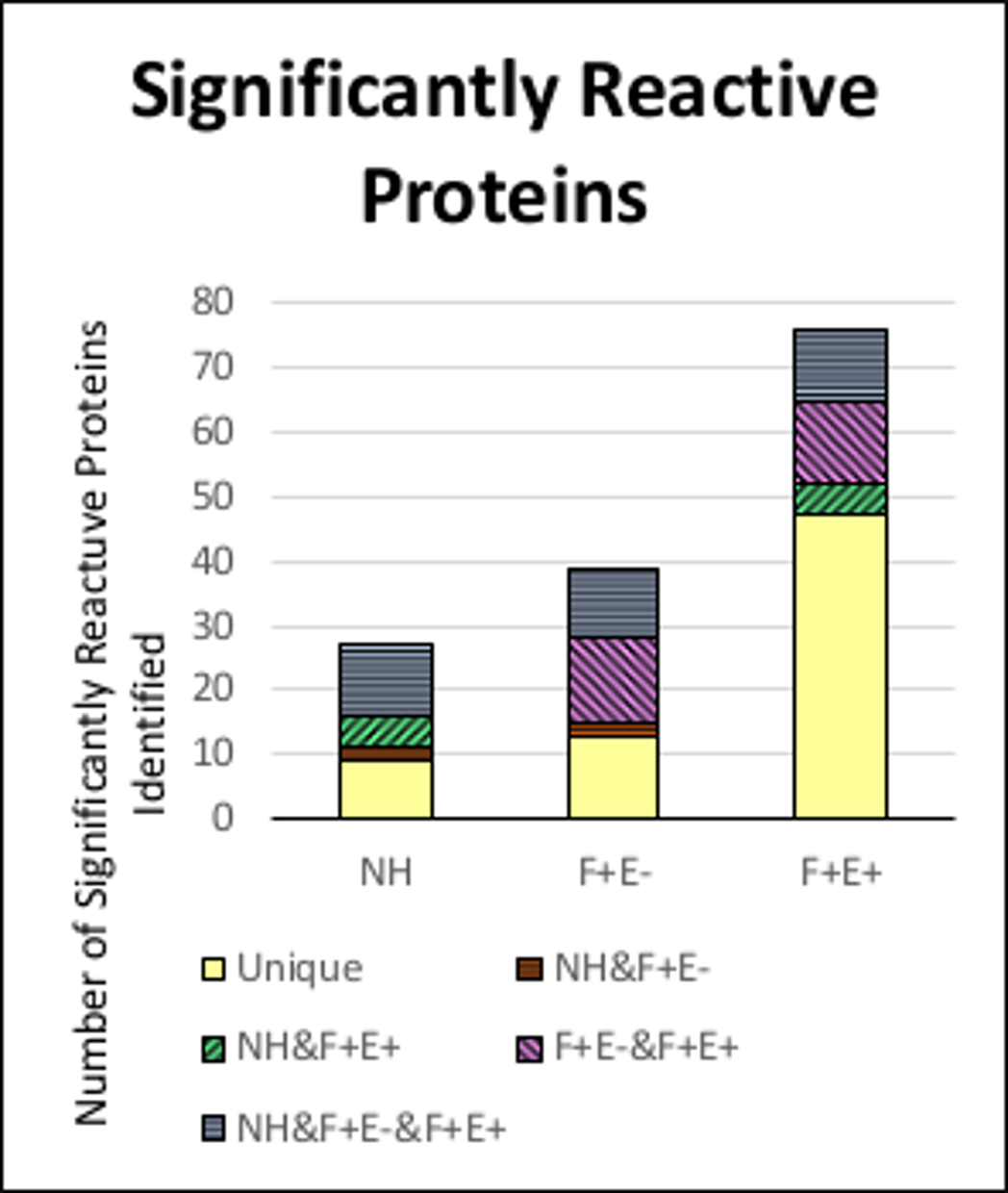

Supplement: S2 Fig — Unique proteins represents proteins the proteins are significantly reactive (P<0.05) proteins identified only in the specific group (NH, F+E-, or F+E+). Shared proteins represent proteins are significantly reactive (P<0.05) proteins identified in two or three groups. (TIFF) [file pone.0184373.s005.tiff]

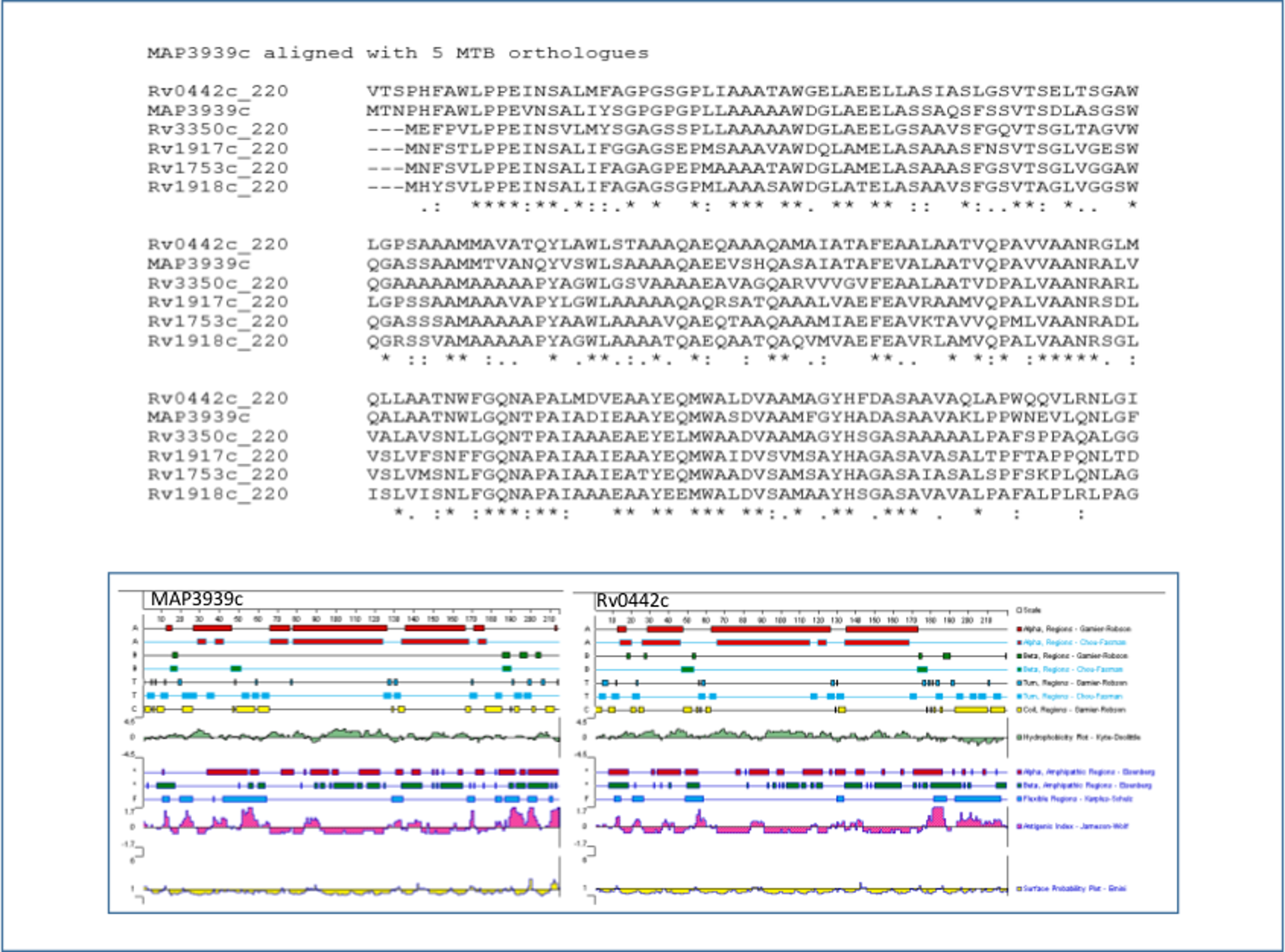

Supplement: S3 Fig — Upper: multiple alignment of MAP3939c with 5 MTB orthologue. As showed in the alignment, there is the highest identity between MAP3939c and Rv0442c. Bottom: similar structure characters between MAP3939c and Rv0442c (Protean of Lasergene, DNAstar, Madison, Wisconsin). (TIFF) [file pone.0184373.s006.tiff]
